# Autotrophic yeast

**DOI:** 10.1038/s41467-024-49586-2

**Published:** 2024-07-16

**Authors:** Jens Nielsen

**Affiliations:** BioInnovation Institute, DK2200 Copenhagen, Denmark and Department of Life Sciences, Chalmers University of Technology, SE41296 Gothenburg, Sweden

**Keywords:** Metabolic engineering, Synthetic biology, Applied microbiology, Experimental evolution

## Abstract

Yeast is a widely used cell factory for the conversion of sugar into fuels, chemicals and pharmaceuticals. Establishing yeast as being autotrophic can enable it to grow solely on CO_2_ and light, and hereby yeast can be used as a wider platform for transition to a sustainable society.

Metabolism is the core of cellular function as it provides the building blocks for biosynthesis of macromolecules, the free energy required for their synthesis, and it ensures balancing of redox in the large number of different reactions occurring within a cell. Over millions of years metabolism has evolved differently in different life forms to enable adaption to the environment where they live. A major event in cellular evolution was establishment of the eukaryotic cell through creating an endosymbiont of a bacterial and archaeal cell. In this endosymbiont the energy machinery of the bacteria, i.e. the tricarboxylic acid and the respiratory system, has evolved to become the main source of energy generation in the eukaryotic cell. In a similar fashion chloroplast evolved from engulfing a cyanobacterium into a non-photosynthetic cell. The concept of complementing metabolic capabilities of different life forms has therefore played an important role in evolution of metabolism.

In a recent *Nature Communication* paper, Gao et al.^1^ demonstrated that this concept can also be applied in the laboratory to generate cells with new capabilities. They first engineered the cyanobacterium *Synechococcus elongatus* to secrete glucose assimilated from photosynthesis. They did this by expressing two genes from the bacterium *Zymomonas mobilis* that encodes for an invertase that degrade sucrose to glucose and fructose and a facilitated transporter that can ensure secretion of glucose. The cyanobacterium will use part of the sugar to generate free energy in the form of ATP required for growth whereas excess sugar will be secreted. To ensure that the yeast and the cyanobacterium could live in endosymbiosis the authors established mutual dependencies. The cyanobacterium was made methionine auxotroph such that when it should live in endosymbiosis with yeast, they will have to acquire methionine needed for growth from yeast. Furthermore, the respiratory system in yeast was inactivated by deletion of *cox2-60*, a key component of mitochondrial respiration. This yeast cannot grow on respiratory carbon sources like glycerol but can grow on fermentative carbon sources like glucose and yeast therefore will be dependent on supply of glucose from the cyanobacterium. By creating an endosymbiont with these two strains, Gao et al. could demonstrate that yeast could grow on CO_2_ as the sole carbon source (Fig. 1A), and they validated that ^13^C-labeled CO_2_ was incorporated into yeast biomass.

**Fig. 1:**
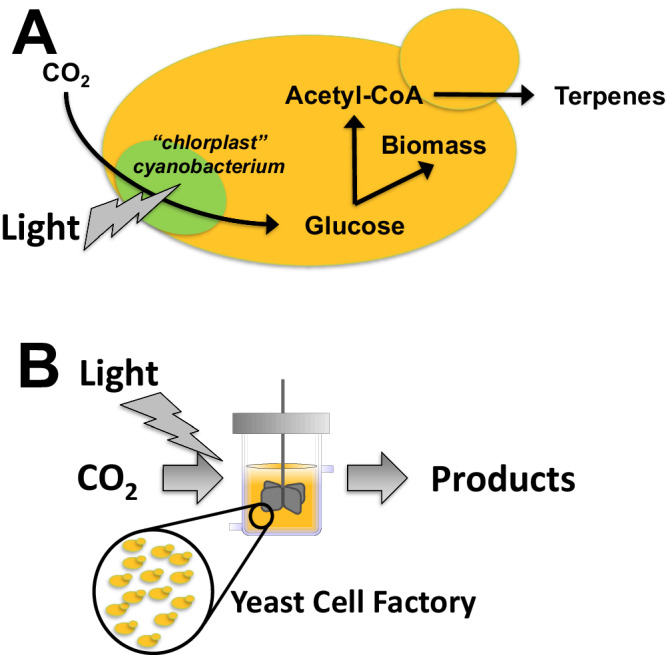
Establishment of yeast as an autotrophic organism that can convert CO_2_ and light into chemicals. **A** By engulfing a cyanobacterium into yeast there was established a synthetic chloroplast that can convert CO_2_ and light into glucose. The formed glucose can be used by the cells to make more yeast cells, i.e. biomass. Part of the glucose can also be recruited for biosynthesis of valuable chemicals, e.g. terpenes. **B** The yeast/cyanobacterium chimera can be used more widely for conversion of CO_2_ and light into a wide range of products, i.e. fuels, chemicals, foods, materials and pharmaceuticals.

Next, Gao et al. engineered the endosymbiont to produce terpenes, more specifically the monoterpene limonene. Limonene can be synthesized from geranyl pyrophosphate (GPP) by expressing two heterologous enzymes in yeast. GPP is one of the end products of the mevalonate pathway and an intermediate in the endogenous pathway leading to sterols, and the mevalonate pathway has earlier been recruited for production of a range of different terpenes, in particular sesquiterpenes used as ingredients in fine fragrances^2^ or as valuable pharmaceuticals like the antimalarial drug artimisinic acid^3^. Monoterpenes has also earlier been produced by yeast from glucose through expressing heterologous plant enzymes in yeast^4^, but direct production of monoterpenes from CO_2_ does represent a potentially far more sustainable route for production of these chemicals as well as many other terpenes (Fig. 1B).

Gao et al. also demonstrated that their concept can be used to make laboratory yeast strains photosynthetic by incorporating their cyanobacteria into such strains. This opens up for a wide range of applications where yeast strains engineered for production of different chemicals can be converted to use CO_2_ as a carbon source. As yeast is used as a cell factory for production of a wide range of chemicals^5,6^, the perspective of the work is quite large as it represents an alternative to earlier approaches where photosynthetic pathways have been engineered into methylotrophic yeast^7^. The work may therefore represent an important step towards establishing so-called third-generation bioprocesses where CO2 is used as carbon source for production of fuels, chemicals, and pharmaceuticals^8^. However, a major requirement for this is to make the process more efficient, such that it can meet key techno-economic requirements for commercially viable processes, and hence it needs to focus on improving titer, rate and yield of the potential future process^9^.
